# Localisation of an Unknown Number of Land Mines Using a Network of Vapour Detectors

**DOI:** 10.3390/s141121000

**Published:** 2014-11-06

**Authors:** Hiba Haj Chhadé, Fahed Abdallah, Imad Mougharbel, Amadou Gning, Simon Julier, Lyudmila Mihaylova

**Affiliations:** 1 University of Technology of Compiègne, rue Roger Couttolenc, Compiègne 60200, France; E-Mail: fahed.abdallah@hds.utc.fr; 2 Lebanese University, Beirut, Lebanon; E-Mail: imadmoug@ul.edu.lb; 3 Department of Computer Science, University College London, WC1E 6BT London, UK; E-Mails: e.gning@ucl.ac.uk (A.G.); s.julier@ucl.ac.uk (S.J.); 4 University of Sheffield, S1 3JD Sheffield, UK; E-Mail: l.s.mihaylova@sheffield.ac.uk

**Keywords:** land mines localisation, advection-diffusion, inverse problem, Bayesian inference, Markov chain Monte Carlo, PCA

## Abstract

We consider the problem of localising an unknown number of land mines using concentration information provided by a wireless sensor network. A number of vapour sensors/detectors, deployed in the region of interest, are able to detect the concentration of the explosive vapours, emanating from buried land mines. The collected data is communicated to a fusion centre. Using a model for the transport of the explosive chemicals in the air, we determine the unknown number of sources using a Principal Component Analysis (PCA)-based technique. We also formulate the inverse problem of determining the positions and emission rates of the land mines using concentration measurements provided by the wireless sensor network. We present a solution for this problem based on a probabilistic Bayesian technique using a Markov chain Monte Carlo sampling scheme, and we compare it to the least squares optimisation approach. Experiments conducted on simulated data show the effectiveness of the proposed approach.

## 1. Introduction

An anti-personnel mine is a device designed to injure or to kill whomever comes into contact with it through direct pressure or a trip-wire [1]. Land mine detection, localisation and clearance is of great importance due to the danger that buried land mines still represent to people all over the world. It is pointed out in [2] that about 50–100 million anti-personnel mines exist in more than 80 countries and that more than 20,000 people are killed or injured every year due to the explosion of buried land mines. Though the Ottawa treaty prohibited in 1997 the use of this weapon, some countries have not signed the treaty, and nearly two million land mines are laid per year. The dangers are particularly acute for those responsible for localising and decommissioning land mines. To limit the number of victims, land mine detection and clearance actions have taken place since the end of the Second World War [3,4]. Historically, the most common method for land mine detection is metal detection. Although this method has proved to be effective with primary land mines, it fails with many modern land mines, which are fabricated from sophisticated non-metallic materials, such as plastic and wood [1], making them invisible to the metal detector. Therefore, many other methods have been developed. These include the use of trained dogs and several physical detection techniques based on ground penetrating radar (GPR), X-ray, infrared (IR) imaging [5,6], neutron activation (TNA) and nuclear quadrupole resonance (NQR) [2,7]. However, a common problem with all these techniques is that the probability of false positives is high [8]. Other approaches employ unmanned vehicles for landmine detection [9]. This technique requires sophisticated and rather expensive equipment and control.

This paper presents a method to address the land mine localisation problem using data collected from a network of wireless sensors capable of detecting the concentration of the explosive chemicals in the air. The explosive chemicals, such as trinitrotoluene (TNT) or dinitrotoluene (DNT), leak out from buried land mines into the surrounding environment and are transported through the air by mechanisms such as advection and diffusion. Sensors for these types of chemical explosive materials exist [10]. By expressing the concentration of the explosive as a function of the land mines' locations and by solving the inverse problem, we will show that the proposed framework is able to detect, locate and find the emission rates of several land mines.

All existing methods for land mine detection consider a known number of sources in a specific region [11,12]. However this information rarely exists for real applications. One original contribution of this paper is to present a solution for an *unknown number* of land mines or vapour-emitting sources. In our method, the objective is to estimate the number of sources and then characterise them. Note that throughout this paper, the term *source characterisation* will refer to determining the sources unknown parameters including their positions and emission rates. Briefly speaking, the set of concentration measurements which have been made by the detection system are grouped in a matrix and a PCA scheme is used in order to determine the number of sources. Once this number is known we will be able to locate explosive sources and to estimate the emission rate of each source. Thus, in difference to most of existing methods, we solve the problem of characterisation of *multiple* anti-personnel land mines.

The localisation problem is modelled through a probabilistic Bayesian approach and a Markov chain Monte Carlo (MCMC) algorithm; namely, that of slice sampling is used in order to sample from the posterior density of interest. This probabilistic approach is tested and compared to an optimisation technique, *i.e.*, the popular least squares approach [13]. The advantages and limitations of both techniques are discussed in detail. There are two main advantages of using a probabilistic approach. First, the solution provided takes on the form of a probability distribution, so the uncertainty on the estimated position can be quantified [14]. Another important advantage of the proposed Bayesian technique is that it overcomes the convergence problems (to local minima) that the least squares approach could face.

The rest of the paper is organised as follows. Section 2 reports some related work. In Section 3, we describe the model used for the transport of the explosive chemical in the air and thus, formulate the direct or forward problem. A matrix of concentration measurements is obtained, and a PCA scheme is used to determine the unknown number of sources in Section 4. In Section 5, we define the inverse problem consisting of locating the land mines and determining their emission rates given concentration measurements from randomly deployed sensors. We first present a probabilistic Bayesian approach for solving the problem; then, we explain how the generic least squares approach can be used in order to solve the sources characterisation problem. Section 6 gives some simulation results and finally Section 7 concludes the paper.

## 2. Related Work

The advances in sensing technologies [8] increase the use of sensor networks in a vast range of applications [12,15,16]. Recently, wireless sensor networks (WSNs) have become popular in source identification applications. In fact, risk management applications in the fields of environment [14,17,18] and security [15,19] rely on data collected from a WSN in order to characterise a source of dispersion, e.g., in the case of an accidental or intentional release of a chemical or biological substance in the air. In [19], an algorithm is derived to detect CO2 leaks at several potential locations at a carbon sequestration site. The aim in [18] is to study the emissions of a number of contaminant sources, located at well-known positions, at a large lead-zinc smelter. In [14,15,17], probabilistic Bayesian approaches are used to determine the unknown position and possibly other model parameters, such as the emission rate and the diffusion coefficient, of a single dispersion source using data collected from a WSN. In [20], a recursive algorithm based on a state space representation of the system is developed to estimate a single diffusion source position and to track its intensity in time using concentration measurements provided by a sensor network. In [21] theoretical results are derived characterising the accuracy of the location estimate of a single gas emitting source using a network of binary sensors. The measurements are quantised and a single bit of information is generated depending on whether the sensed value is lower or higher than some threshold. In [22] a computation method is proposed to overcome the difficulties associated with the choice of an adequate dispersion model and the calculation of the likelihood function in a Bayesian framework in order to solve the problem of localisation of a source of toxic release.

The idea of using a sensor network for land mine localisation is addressed in [11,23], but both consider the case of a single land mine. In [23], the problem of localisation of a single land mine is considered using an analytical solution of the inverse problem, not taking into account a model or measurement noise. In [11], a maximum likelihood estimation algorithm is derived in order to locate a single land mine and find its emission rate. The performance of the estimator is evaluated by computing the Cramer-Rao bound.

The case of parameter estimation for multiple sources is briefly addressed in [24] and [25] in a Bayesian probabilistic and an optimisation least squares frameworks and for a known number of sources. A more difficult case when the number of sources is unknown is addressed in [26–28]. In [26,28], the problem is formulated as a generalised parameter estimation problem, where the number of sources is included in the vector of unknown parameters. This approach implements a reversible-jump MCMC algorithm and requires intensive computations since the dimensionality of the unknown parameters' vector is variable. In [27], Yee formulates the problem of characterising an unknown number of sources as a model selection problem. While this approach is less complex than the previous one, it is also computationally demanding.

The application of interest in this paper is the localisation of multiple anti-personnel land mines using a WSN, where we consider the case of an unknown number of sources. In our method, we propose to use PCA on a matrix of concentration measurements in order to determine the unknown number of land mines. The use of this strategy makes the problem of localisation less complex and more efficient. The localisation problem is addressed after in a probabilistic Bayesian framework, so that the solution provided takes on the form of a probability distribution. The uncertainty on the estimated position can thus be quantified [14], rather than approximated, e.g., by computing the Cramer-Rao bound, as in [11]. The probabilistic approach is also compared to the optimisation least squares technique.

## 3. The Forward Problem

The forward model is used to compute an estimated concentration of the explosive chemical at a certain location given a vector of parameters consisting of land mine locations, emission rates and environmental conditions, such as the diffusivity of the air and the wind velocity. It describes the transport of the explosive chemical emitted by the land mines due to the advection and diffusion processes. Note that numerical solutions for modelling the transport of TNT emanating from land mines [29] were proposed. In this paper, we model a land mine as a point source placed on an impermeable planar surface and diffusing an explosive chemical, such as the TNT, at a constant rate [12], and an analytical model for the transport of the explosive vapours is used.

Let us first consider the case of a single land mine placed at position **r***_s_* = (*x_s_*, *y_s_*, *z_s_*)*^T^* in the plane *z* = 0. The source emits the explosive chemical with a constant rate *Q* (g/s) starting at time *t*_0_. The wind velocity is modelled as a constant vector directed along the x-axis and of a constant magnitude *V* (m/s). The differential equation [12,30] governing the variation of the concentration *C*(*t*, **r**) of the explosive chemical at time *t* ≥ *t*_0_ and at position **r** = (*x*, *y*, *z*)*^T^* in the semi-infinite medium *z* ≥ 0 is given by:

(1) $$\begin{array}{cc} \frac{\partial C}{\partial t} & -K\left(\frac{\partial^{2}C}{\partial x^{2}}+\frac{\partial^{2}C}{\partial y^{2}}+\frac{\partial^{2}C}{\partial z^{2}}\right)+V\frac{\partial C}{\partial x}\\ & =2Q.u\left(t-t_{0}\right).\delta \left(x-x_{s}\right).\delta \left(y-y_{s}\right).\delta \left(z-z_{s}\right) \end{array}$$

where *K* (*m*^2^/*s*) is the air diffusion coefficient; *δ*(.) denotes the Dirac delta function and *u*(*t* − *t*_0_) refers to the step function vanishing for *t* < *t*_0_ and equal to unity for *t* ≥ *t*_0_. The solution [30] for *t* ≥ *t*_0_ at position **r** given the initial condition of zero concentration for all **r** is given by:

(2) $$\begin{array}{c} C\left(Q,{\text{r}}_{s},\text{r},t,t_{0}\right)=\frac{Q}{\pi^{\frac{3}{2}}Kd}\text{exp}\left(\frac{V\left(x-x_{s}\right)}{2K}\right)\\ \times \int_{\frac{d}{2\sqrt{K\left(t-t_{0}\right)}}}^{\infty }\text{exp}\left(-u^{2}-\frac{V^{2}d^{2}}{16K^{2}u^{2}}\right)du \end{array}$$

where *d* = *d*(**r***_s_*, **r**) = ‖**r***_s_* − **r**‖ is the Euclidean distance between positions **r** = (*x*, *y*, *z*)*^T^* and **r***_s_* = (*x_s_*, *y_s_*, *z_s_*)*^T^*.

Under the assumptions of constant *V* and *Q*, and after a sufficiently long time (*t* → ∞), a stationary concentration profile [12,30], given by the following concentration, is established:

(3) $$C_{\infty }\left(Q,{\text{r}}_{s},\text{r}\right)=\frac{Q}{2\pi Kd}\text{exp}\left(-\frac{V.\left(d-\left(x-x_{s}\right)\right)}{2K}\right)$$

In the remaining sections, the sources and the sensors are considered to be in the same plane *z* = 0, and we omit thus the third coordinate *z* in all position vectors.

**Figure 1.**
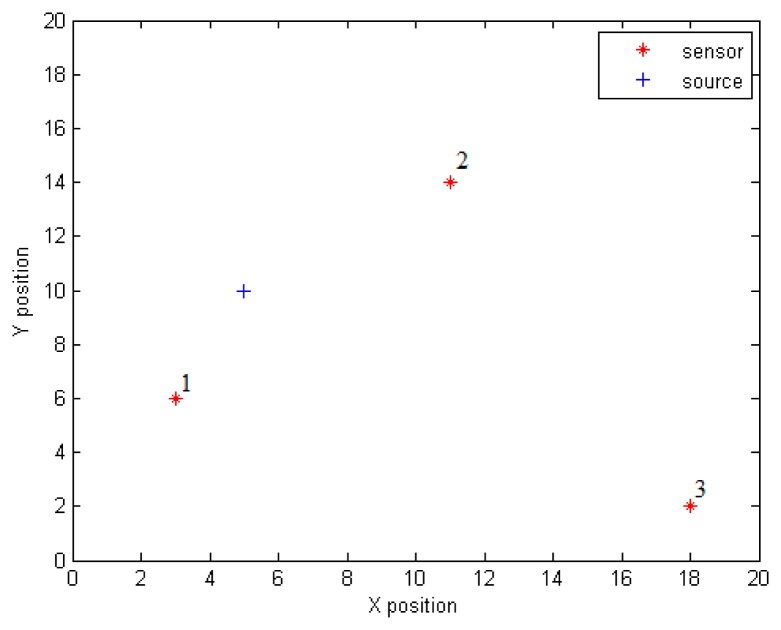
An illustrative scenario with a single source and three sensors.

**Figure 2.**
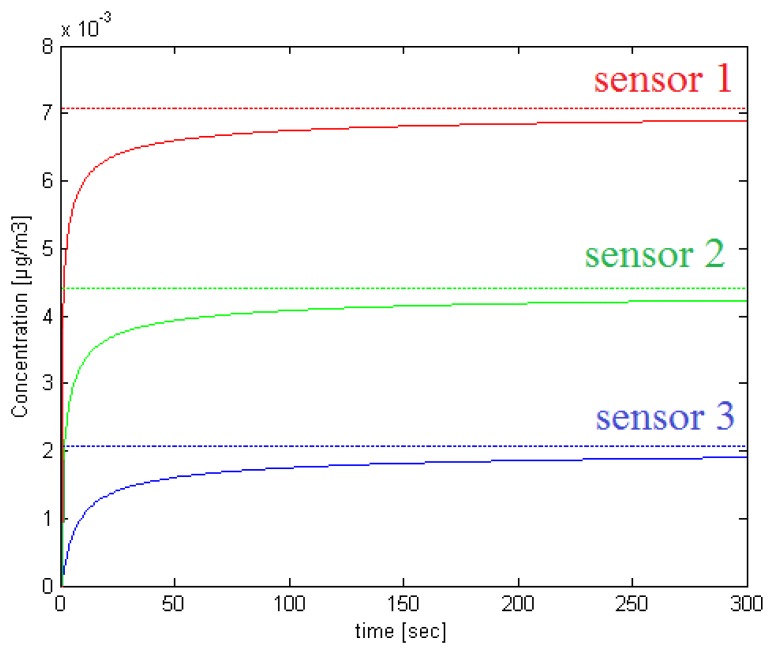
Concentration profiles are given in solid line at the sensors positions for the scenario given in Figure 1. Dotted lines show the stationary concentrations.

Figures 1 and 2 show respectively an illustrative scenario with one source and three sensors in a 20 m × 20 m planar region, and the variation of the concentration at the three sensors positions as given in Equation (2) for *Q* = 5*μg*/*s*, *K* = 25m^2^/*s* and *V* = 5*cm*/*s*. The stationary concentrations are also shown in dotted lines. The graph shows that the concentration change within 5 min reaches 97.50, 95.96 and 91.40 percent of the stationary concentration that would be established at the positions of Sensors 1, 2 and 3, respectively.

Let us consider now the case of *N* sources, *i.e.*, land mines. The *i* – *th* source, denoted as *S_i_*, *i* = 1, …, *N*, is at position **r***_S_i__* = (*x_S_i__*, *y_S_i__*)*^T^* and has a constant emission rate denoted by *Q_i_*. The *resultant* stationary concentration [18] at position **r** = (*x*, *y*)*^T^* is given by:

(4) $$C_{r}\left({\left\{Q_{i},{\text{r}}_{s}\right\}}_{i=1}^{N},\text{r}\right)=\sum_{i=1}^{N}C_{\infty }\left(Q_{i},{\text{r}}_{s_{i}},\text{r}\right)$$

Consider *M* sensors which are placed at known positions **r***_j_* = (*x_j_*, *y_j_*)*^T^*, *j* = 1, …, *M*. Referring to Equations (3) and (4), the estimated concentration at position **r***_j_* of sensor *j* can be expressed as:

(5) $$C_{rj}\left({\left\{Q_{i},{\text{r}}_{s_{i}}\right\}}_{i=1}^{N},{\text{r}}_{j}\right)=\sum_{i=1}^{N}Q_{i}\times g_{ji}\left({\text{r}}_{s_{i}},{\text{r}}_{j}\right)$$

where

(6) $$g_{ji}\left({\text{r}}_{s_{i}},{\text{r}}_{j}\right)=\frac{1}{2\pi Kd_{j}^{i}}\text{exp}\left(-\frac{V.\left(d_{j}^{i}-\left(x_{j}-x_{s_{i}}\right)\right)}{2K}\right)$$

and $d_{j}^{i}=\left\|{\text{r}}_{s_{i}}-{\text{r}}_{j}\right\|.$

Let **P** denote the vector of parameters of the sources, *i.e.*, source' positions and emission rates. For *N* land mines,

$$\text{P}={\left[{\left\{x_{s_{i}},y_{s_{i}},Q_{i}\right\}}_{i=1}^{N}\right]}^{T}$$

The concentration measurements provided by the sensor network are grouped in an array denoted by ${\text{Y}}^{m}={\left[C_{1}^{m},\ldots ,C_{M}^{m}\right]}^{T}.$

Let ${\text{Y}}^{t}={\left[C_{1}^{t},\ldots ,C_{M}^{t}\right]}^{T}$ denote the vector of true concentrations at positions **r***_j_*, and ${\text{E}}^{m}={\left[e_{1}^{m},\ldots ,e_{M}^{m}\right]}^{T}$ a vector of measurements error, thus:

(7) $$C_{j}^{m}=C_{j}^{t}+e_{j}^{m},\;j=1,\ldots ,M$$

Furthermore, if ${\text{Y}}^{e}={\left[C_{1}^{e},\ldots ,C_{M}^{e}\right]}^{T}$ refers to the vector of *estimated* concentrations at **r***_j_*, *i.e.*, the concentrations obtained by resolving the forward problem according to Equation (4), and ${\text{E}}^{e}={\left[e_{1}^{e},\ldots ,e_{M}^{e}\right]}^{T}$ a vector of model error, then:

(8) $$\begin{array}{cc} C_{j}^{e}=C_{j}^{t}+e_{j}^{e}, & j=1,\ldots ,M \end{array}$$

Based on Equations (7) and (8), we can write:

(9) $$\begin{array}{cc} C_{j}^{m}=C_{j}^{e}+e_{j}, & j=1,\ldots ,M \end{array}$$

where *e_j_* denotes the quantity characterising the difference between measured and modelled/estimated concentrations. Equivalently, in vector notation:

(10) $$\begin{array}{c} {\text{Y}}^{m}={\text{Y}}^{t}+{\text{E}}^{m}\\ {\text{Y}}^{e}={\text{Y}}^{t}+{\text{E}}^{e} \end{array}$$

and:

(11) $${\text{Y}}^{m}={\text{Y}}^{e}+\text{E}$$

where **E** refers to an error. Based on (4) and (10), we can define an operator **F**, such that:

(12) $${\text{Y}}^{m}=\text{F}\left(\text{P},\text{A}\right)$$

where **A** is a constant vector grouping information about the environment, sensors characteristics and model applicability, e.g., wind velocity, air diffusion coefficient and noise variances.

**Figure 3.**
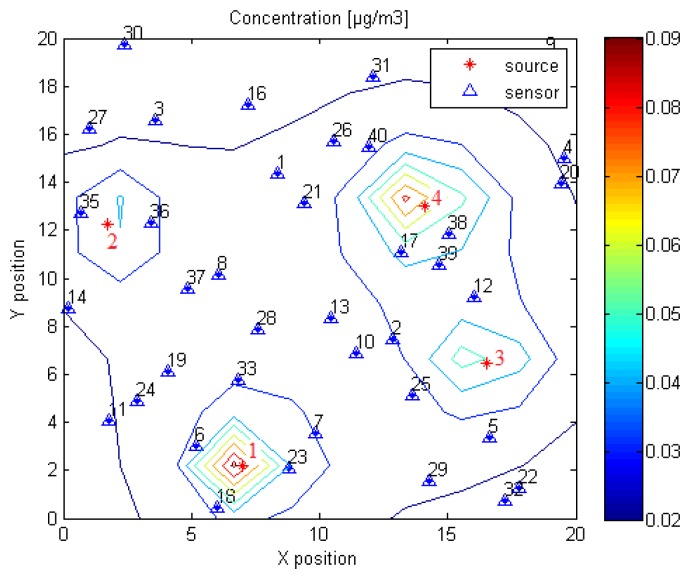
Illustration of the forward problem.

Figure 3 shows the stationary concentration profile of the emitted chemical determined using Equation (4) for a scenario with *N* = 4 sources and *M* = 40 sensors, which are randomly deployed in a 20 × 20 *m*^2^ planar region. Emission rates are randomly fixed to *Q*_1_ = 8.01 *μg*/*s*, *Q*_2_ = 5.38 *μg*/*s*, *Q*_3_ = 5.87 *μg*/*s* and *Q*_4_ = 9.39 *μg*/*s*.

## 4. Determining the Unknown Number of Sources

In this section, we use the principal component analysis (PCA) in order to determine the number of sources based on a set of measurements. PCA [31] is a popular statistical method that has been widely applied in the analysis of multidimensional data sets, which are usually represented by tables of observations of many possibly inter-correlated variables. Since the information provided by these variables is often redundant, PCA attempts to replace the original set of variables by a smaller number of new variables, called principal components, without losing too much information. This technique considers that the new variables are linear combinations of the original ones and that they are linearly uncorrelated.

Mathematically, PCA transforms the data to a new coordinate system, such that the original set of observations is expressed in terms of the principal components. A technique for performing PCA is to compute the covariance matrix calculated from the available measurements and then to determine its eigenvalues. The corresponding normalised eigenvectors, ordered according to decreasing eigenvalues, define the new coordinate system. A smaller dimensional coordinate system, which is supposed to conserve most of the information, can be obtained by only retaining the eigenvectors associated with the largest eigenvalues.

**Figure 4.**
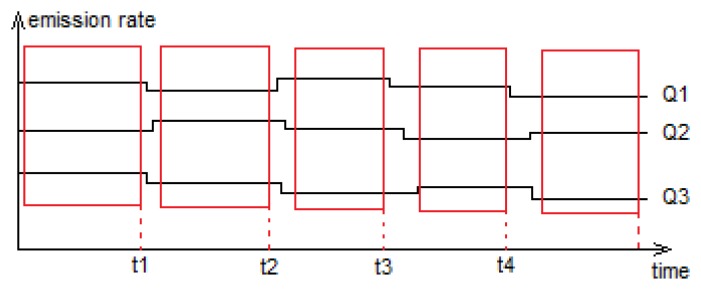
The emission rates are piecewise constant, and a series of stationary concentration profiles are established.

In our application, *M* sensors are randomly deployed in a region of interest where the unknown number of vapour-emitting sources is denoted by *N*. Each sensor provides measurements of the concentration of the explosive material emitted by the sources and transported to the sensor's position due to advection and diffusion processes. Supposing that concentration measurements are taken on each sensor at *T* time instants, the measurements are grouped in an *M* × *T* matrix **C** = (*C_jt_*), *j* = 1, …, *M*, *t* = 1, …, *T*, where the *j* – *th* row groups the concentration measurements recorded by the *j* – *th* sensor at different time instants and the *t* – *th* column groups measurements recorded by all *M* sensors at time instant *t*. Since the land mines emissions are likely to change slowly over time, we model these emissions using piecewise constant functions. Referring to Figure 2 of the previous section, the stationary concentration profile is established within only a few minutes; thus, the recorded concentration measurements can be considered as stationary concentrations established for the constant emission rates between time instants *t* − 1 and *t* similarly to what was suggested in [17]. Figure 4 recapitulates the assumptions we make. It illustrates the variation of the emissions of three sources and indicates, using red boxes, the regions where stationary concentration profiles are established. The measurements grouped in matrix **C** are, for instance, taken at time instants *t*_1_, *t*_2_, *etc*. Note that these measurements can be chosen by simply examining the signals provided by the sensors and detecting stationary points, *i.e.*, the steady-state concentration values.

In the following, *Q_it_* denotes the emission rate of source *i*, *i* = 1, …, *N*, considered as constant between time instants *t* − 1 and *t*. We consider an *N* × *T* matrix **Q** = (*Q_it_*) grouping the emission rates of the sources, where each row *i* is associated with source *i* and each column *t* groups the emissions of the sources at time *t*. Let **G** = (*g_ji_*) be an *M* × *N* matrix grouping the factors *g_ji_*(**r***_S_i__*, **r***_j_*) associated with source *i* and sensor *j*, as in Equation (6). If **C***_t_* denotes the *t* – *th* column of matrix **C**, *i.e.*, the vector of concentration measurements provided by the sensors at time *t* and **Q***_t_* denotes the *t* – *th* column of matrix **Q**, *i.e.*, the vector of sources emission rates at time *t*, referring to Equation (5), we have in matrix notation:

(13) $$\begin{array}{cc} {\text{C}}_{t}=\text{G}\;\times \;{\text{Q}}_{t}, & t=1,\ldots ,T \end{array}$$

Taking into account an additive noise, as in Equation (9), we obtain:

(14) C=G×Q+ϵ

where ***ϵ*** = (*e_jt_*), 1 ≤ *j* ≤ *M*, 1 ≤ *t* ≤ *T* is an additive noise matrix.

Equation (14) shows that the concentrations measured on the sensors are linear combinations of the sources emission rates. In our application, the only available information is the matrix of concentrations **C**. Performing the PCA technique on matrix **C** and retaining only the eigenvalues, which are larger than some threshold *λ_th_*, allows to transform the data to a new coordinate system whose dimension is *d* ≤ *M*. An ideal choice of the threshold would recover precisely the number of sources (*d* = *N*). The condition *d* ≤ *M* implies that the number of sensors cannot be less than the number of mines.

In our simulations, the number of concentration records is fixed to *T* = 10, and at each time instant, the emission rates of the sources are drawn uniformly from the interval [5 10]*μg*/*s*. The sources and the sensors are randomly placed in a 20 m × 20 m region.

In order to develop the relationship when *N* ≤ *M*, we studied what is the minimal number of sensors that can detect the number *N* of the true hidden sources using the PCA technique for the simulation conditions described above. In our study, we fixed first the number *N* of sources and initiated *M* = *N*. A hundred source-sensor configurations, obtained by randomly deploying *N* sources and *M* sensors in a 20 m × 20 m region, are generated. The PCA technique is tested on the different configurations, and the number of sensors is iteratively increased until the true number of sources is obtained for at least 90% of the configurations. The results are given below in Table 1. Using these simulations, an adequate threshold *λ_th_* = 10^−7^ is empirically determined. Note that for *T* = 10 and for the simulation conditions, we fixed the maximal detectable number to be *N* = 8 sources.

## 5. Definition of the Inverse Problem for Source Characterisation

The *inverse problem* can be defined as the process of inferring causes, conditioned on knowledge of the effects, as opposed to the forward or direct problem, allowing to determine the effects knowing the causes. An example of an inverse problem, which will be of main concern in this paper, is the one of parameter identification [13]. The corresponding forward problem consists of determining the output of a system knowing the system's parameters. A practical difficulty in the study of an inverse problem is that it is often ill-posed [14], meaning that an inverse transformation of the direct model may not exist, may not be unique, and might be unstable. Amongst the most general and popular techniques are least squares approach and regularisation techniques. The key is to reformulate the inverse problem as an optimisation problem, usually consisting of minimising a functional error between actual measurements and predicted ones obtained by resolving the direct problem. Probabilistic approaches can also be used to address the problem of parameter identification. A primary advantage of probabilistic methods is that the solution takes on the form of a probability distribution rather than a point solution, optimal in terms of a given criterion [14].

Referring to Equation (12) where **A** is a constant vector, we can define an operator **H**, such that:

(15) $${\text{Y}}^{m}=\text{H}\left(\text{P}\right)$$

and introduce the inverse source characterisation problem as finding:

(16) $$\text{P}\underline{\approx }{\text{H}}^{-1}\left({\text{Y}}^{m}\right)$$

The exact solution for Equation (16) is usually not tractable. This is primarily due to the existence of model and measurement noises. In other words, the forward model is not perfectly known. Furthermore, due to the commutative property of the addition in Equation (4), the solution is not unique; for instance, in the case of two land mines, *i.e.*, *N* = 2, if **P**_1_ = [*x̃_s_*_1_, *ỹ_s_*_1_, *Q̃*_1_, *x̃_S_*_2_, *ỹ_S_*_2_, *Q̃*_2_]*^T^* is a solution to the inverse problem, then **P**_2_ = [*x̃_s_*_2_, *ỹ_s_*_2_, *Q̃*_2_, *x̃_S_*_1_, *ỹ_S_*_1_, *Q̃*_1_]*^T^* is also a solution. In general, any parameter vector obtained by only permuting the labels of the sources in a solution vector is also a solution. The estimation problem is thus over a set of land mines.

### 5.1. Bayesian Inference for Solving the Inverse Problem

In this section, the inverse problem is solved within a probabilistic Bayesian framework. Based on Equation (12), the problem consists in determining **P** having the vector of concentration measurements **Y***^m^* and some prior knowledge gathered in the constant vector **A**. In a Bayesian framework, this refers to finding the posterior distribution *p*(**P**|**Y***^m^*, **A**). According to Bayes theorem:

(17) $$p\left(\text{P}|{\text{Y}}^{m},\text{A}\right)=\frac{p\left({\text{Y}}^{m}|\text{P},\text{A}\right)p\left(\text{P}|\text{A}\right)}{p\left({\text{Y}}^{m}|\text{A}\right)}$$

where *p*(**Y***^m^*|**P**, **A**) is the measurement likelihood, *p*(**P**|**A**) is the prior distribution and *p*(**Y***^m^*|**A**) is the evidence. The evidence measures the suitability of the model (depending on the number of sources) to the available data [14]. The evidence values are calculated and compared for different models in order to determine the most probable number of sources. The higher the evidence, the better the model can predict the data. Since in our method, the number of sources is determined *a priori* as described in Section 4, the evidence is considered as a normalisation factor.

Let us consider a bounded domain denoted as Ω for the land mine locations, *i.e.*, the sources lie within a bounded region [*x_min_ x_max_*] × [*y_min_ y_max_*], and the emission rates are also bounded within lower and upper bounds, *Q_i_* ∈ [*Q_min_ Q_max_*], *i* = 1, … *N*. Choosing a non informative distribution for the prior, *i.e.*, a uniform pdf, we can write:

(18) $$p\left(\text{P}|{\text{Y}}^{m},\text{A}\right)\propto 1_{\text{P}\in \Omega }\;p\left({\text{Y}}^{m}|\text{P},\text{A}\right)$$

where 1**_P_**_∈Ω_ denotes the indicator function taking on a value 1 if **P** ∈ Ω and 0 if not. Additionally, if the measurement and model noises are assumed to be white and Gaussian, *i.e.*:

$$\begin{array}{l} e_{j}^{m}∼N\left(0,\sigma_{m,j}^{2}\right),\\ e_{j}^{e}∼N\left(0,\sigma_{e,j}^{2}\right),\;j=1,\ldots ,M \end{array}$$

then, it can be shown that:

(19) $$p\left({\text{Y}}^{m}|\text{P},\text{A}\right)\propto \text{exp}\left[-\frac{1}{2}\sum_{j=1}^{M}\frac{{\left(C_{j}-C_{j}^{e}\left(\text{P}\right)\right)}^{2}}{\sigma_{m,j}^{2}+\sigma_{e,j}^{2}}\right]$$

Sampling directly from this distribution is difficult, and approximate numerical techniques must be used. A widely used approach for estimating the properties of the posterior distribution given in (18) is to perform Markov chain Monte Carlo (MCMC) sampling [32]. In MCMC algorithms, samples are drawn from the target distribution in the form of a Markov chain where each sample depends on the previous one in the chain. The earliest MCMC algorithm is the random walk Metropolis (RWM) [33]. Its basic principle is to sample a candidate value from a proposal distribution depending on the current position of the chain. The candidate is then accepted or rejected according to the Metropolis acceptance probability as will be defined using the following example. Consider sampling from a pdf π(.). If **x***_i_*_−1_ denotes the current state of the Markov chain, a trial state **z** is sampled according to **z** = **x***_i_*_−1_ + **u**, *where* **u** ∼ *N*(0, Σ), for instance, and Σ denotes a covariance matrix. The candidate **z** is accepted or rejected according to the Metropolis acceptance probability *a* given by:

$$a\left({\text{x}}_{i-1},\text{z}\right)=\{\begin{array}{l} \min\;\left[\frac{\pi \left(\text{z}\right)}{\pi \left({\text{x}}_{i-1}\right)},1\right]\;if\;\pi \left({\text{x}}_{i-1}\right)>0\\ 1\;if\;\pi \left({\text{x}}_{i-1}\right)=0. \end{array}$$

If the candidate is accepted, the chain moves to **x***_i_* = **z**; otherwise, the chain remains at **x***_i_* = **x***_i_*_−1_. The procedure only requires the choice of a proposal function *f*(.).

While the early RWM algorithm requires the proposal distribution *f* to be symmetric, *i.e.*, *f*(**x***_i_*_−1_, **z**) = *f(***z**,**x***_i_*_−1_), the Metropolis-Hastings (MH) algorithm [33] generalises the approach to non-symmetric proposals. Obviously, the choice of the proposal distribution is crucial to the algorithm convergence. Thus, several procedures were proposed in order to improve the algorithm's convergence. These include, for instance, the adaptive Metropolis algorithm [34], the differential evolution Markov chain Monte Carlo (DE-MC) [35] and the differential evolution adaptive Metropolis (DREAM) algorithm [36]. Another class of MCMC sampling techniques is the slice sampling technique [37] and will be used in this paper in order to draw samples from the posterior distribution given in Equation (18).

The slice sampling algorithm relies on the observation that sampling from a probability distribution, e.g., *π*(.) in the case of a univariate distribution, can be done by drawing samples uniformly from the region under the plot of *π*(.) [37]. It has an advantage over other MCMC methods, such as the Gibbs sampler and the RWM, in that the magnitude of the changes made to move from one element to the next in the chain is chosen adaptively.

**Figure 5.**
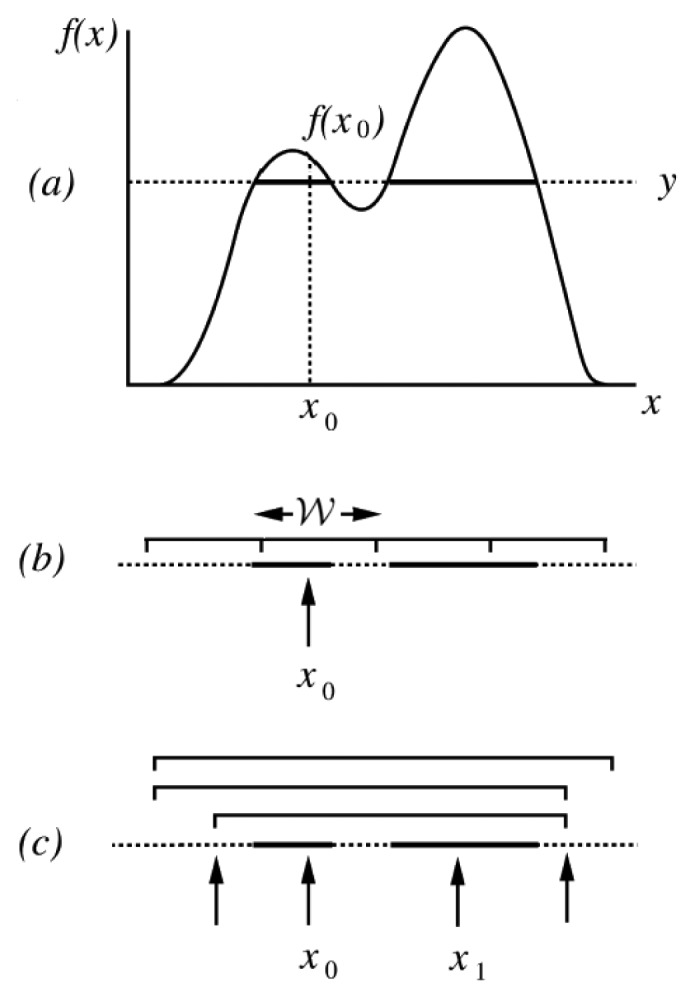
Slice sampling. Adapted from [37].

Figure 5 illustrates the operations of the slice sampling algorithm in the case of a univariate target distribution *π*(.). The procedure requires only the knowledge of a function *f*(.) that is proportional to *π*(.). It operates iteratively in three steps:

(a) Starting from the current position of the chain, denoted as *x*_0_, and such that *f*(*x*_0_) > 0, draw a value *y* uniformly from the interval [0, *f*(*x*_0_)]. The horizontal slice defined by *y* consists of the values of *x* for which *f*(*x*) > *y* (see Figure 5a).
(b) Find an interval around *x*_0_ comprising the majority, or the totality, of the slice defined in (a). Several methods can be used at this step. The approach adopted here (and illustrated in Figure 5b) is called “stepping-out”. It requires fixing, *a priori*, an interval width 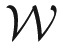 and operates as follows: first, set an interval of width 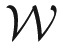 randomly around *x*_0_. Then iteratively expand this interval in steps of size 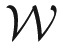 and stop when both interval ends become outside the slice {*x*, *f*(*x*) > *y*}.
(c) Draw a value *x*_1_ from the part of the slice that is within the interval determined in (b). The technique used here is referred to as “shrinkage” (see Figure 5c) because it picks points uniformly from the determined interval, shrinks this last using points that are outside the slice, and stops whenever finding a point inside it.

Slice sampling can also be used to sample from multivariate distributions. This can be done by updating each variable in turn. It is useful though to note that slice sampling methods, which update all variables of a multivariate distribution simultaneously, do exist [37].

### 5.2. The Least Squares Technique for Source Characterisation

In this section, we formulate the source characterisation problem as an optimisation problem and propose to solve it using the least squares (LS) approach. LS is a popular method for solving the inverse problems [13]; it seeks an optimal point solution usually by minimising a quadratic error or cost function between actual measurements and synthetic ones estimated using the forward model.

Referring to Equation (11), we propose to solve the inverse source characterisation problem by minimising over **P**, the vector of unknown parameters, the functional:

(20) $$J={\left({\text{Y}}^{m}-{\text{Y}}^{e}\left(\text{P}\right)\right)}^{T}\left({\text{Y}}^{m}-{\text{Y}}^{e}\left(\text{P}\right)\right)$$

The solution is thus given by:

(21) $${\text{P}}^{\text{opt}}=\underset{\text{P}}{\arg\min}J$$

Despite the wide applicability, the ease of use and ease of understanding associated with the least squares technique, this method presents a well-known problem: it is sensitive to the convexity of the cost function [30] and can converge to local optima, thus diverging from the true solution.

**Figure 6.**
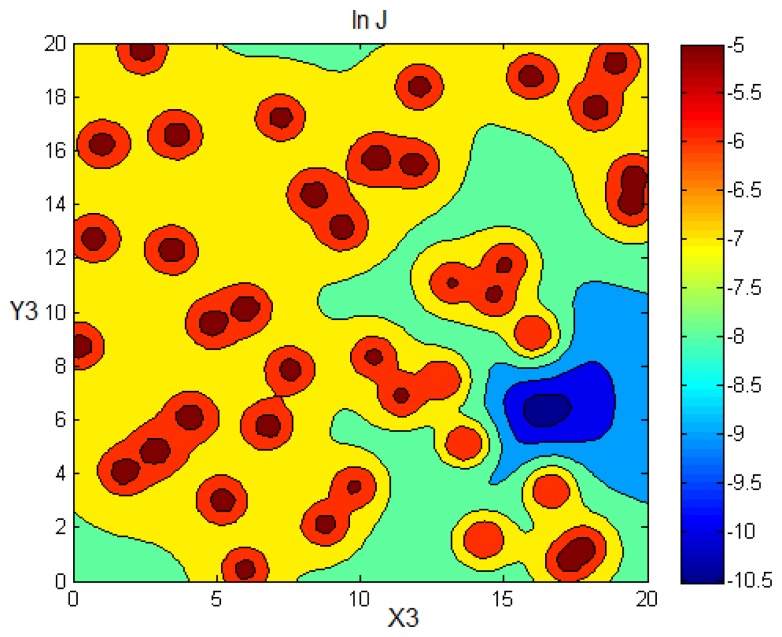
Functional to be minimised in the logarithmic scale.

Figure 6 illustrates the variation of the criterion *J* in terms of Source 3 coordinates for the scenario shown in Figure 3. The values of *J* are calculated as a function of (*x_S_*_3_, *y_S_*_3_) after fixing the remaining unknown parameters, *i.e.*, [*x_s_*_1_, *y_s_*_l_, *x_S_*_2_, *y_S_*_2_, *x_S_*_4_, *y_S_*_4_, *Q*_1_, *Q*_2_, *Q*_3_, *Q*_4_]*^T^*, to their true values. Note that the functional *J* is not convex and presents local maxima at the sensors' positions. This will cause the convergence of the least squares search algorithm to a local minimum, as we show in the next section, where we test the least squares technique on a simulated scenario and compare its performance to the probabilistic Bayesian approach introduced above.

## 6. Simulation Results

In this section, we consider the problem of the localisation of *N* = 4 land mines by randomly deploying *M* = 40 sensors in a 20 × 20 *m*^2^ planar region according to the scenario shown in Figure 3. Model and measurement noises are considered to be white Gaussian with an identical standard deviation equal to 0.001 *μg*/*m*^3^. We fixed the wind velocity to *V* = 5 *cm*/*s* and the air diffusion coefficient to *K* = 25 *m*^2^/*s*, as in [11].

First, a matrix of concentration measurements is obtained as described in Section 4 and a principal component analysis is conducted. The largest eigenvalues are λ_1_ = 0.1116 × 10^−3^, λ_2_ = 0.0751 × 10^−3^, λ_3_ = 0.0345 × 10^−3^, λ_4_ = 0.0159 × 10^−3^; their number is equal to four, which is also the number of the sources. The sum of the remaining eigenvalues is 4.1672 × 10^−8^. Thus, we were able in the first step to successfully determine the number of sources in the considered region. The next step is to localise the sources given concentration measurements.

Table 2 shows the true values of the unknown parameters to be determined; these are the sources positions and emission rates.

### 6.1. Probabilistic Bayesian Approach

The probabilistic Bayesian approach as described in Section 5.1 is tested first. The slice sampling scheme was used in order to draw *N_p_* = 4, 000 particles/samples from the posterior distribution defined in Section 5.

**Figure 7.**
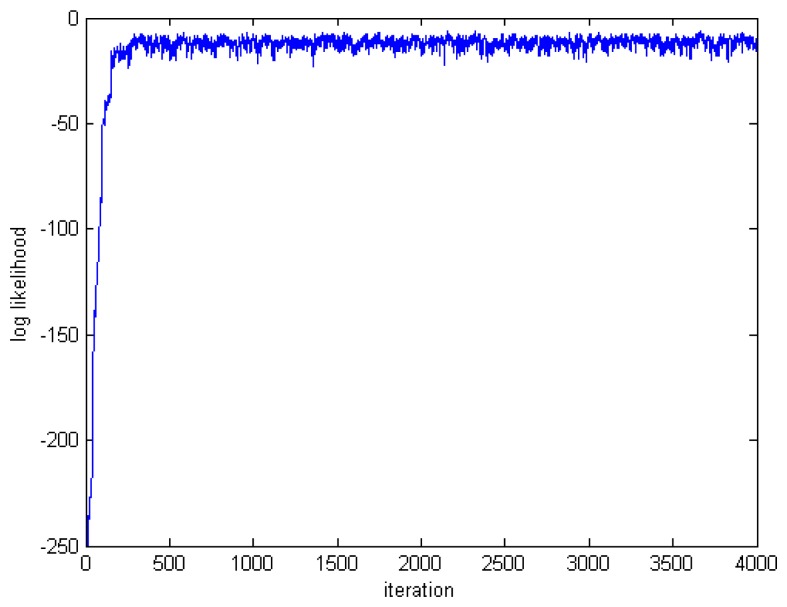
Variation of the log-likelihood in the Markov chain.

**Figure 8.**
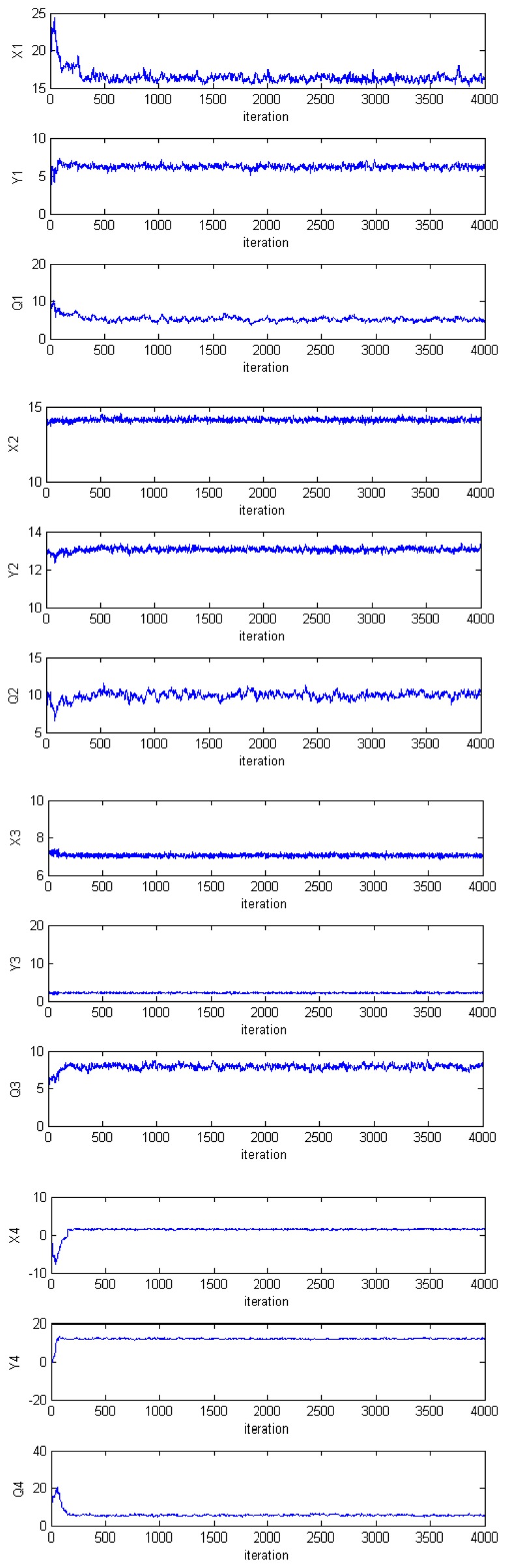
Evolution of the Markov chain.

Figures 7 and 8 show, respectively, the variation of the log-likelihood of the samples and the evolution of the Markov chain through the iterations. The dimension of the parameter vector is 12.

Note on the graphs that there is a transition phase (where the samples likelihood is low) before the chain converges to the posterior distribution of interest. This phase is referred to as the burn-in. In theory, the effect of the initial values tends to zero if the Markov chain is run for an infinite amount of time. In practice, however, an infinite number of samples cannot be drawn, so it is generally assumed that only after a certain number of iterations, the chain reaches the target distribution. Thus, in order to minimise the effect of initial values on the posterior inference, an initial portion, *N_burn_*, of a Markov chain samples is discarded and the remaining samples are used to estimate the properties of the posterior distribution. The number *N_burn_* of the iterations that will be discarded is called the burn-in number.

**Figure 9.**
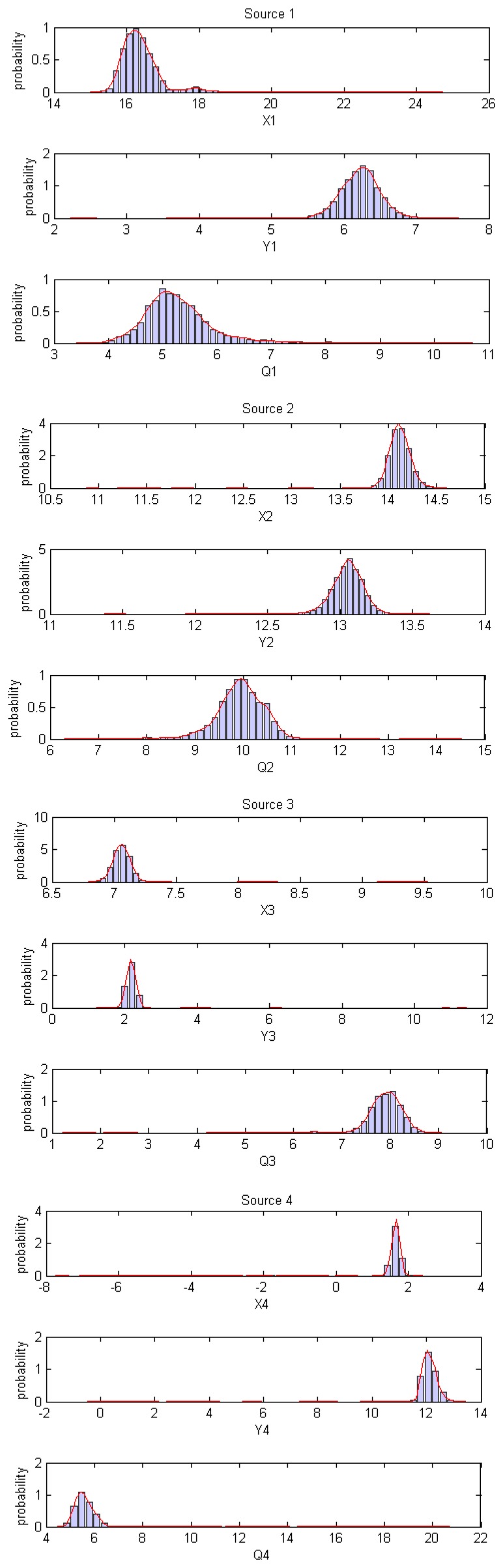
Sample empirical distributions.

Figure 9 shows the normalised histograms of the samples corresponding to the different parameters. The empirical distributions of the parameters are also estimated (using kernel density estimation KDE) and shown in red on the same graphs. Note that the empirical pdfs are centred near the true values of the parameters.

**Figure 10.**
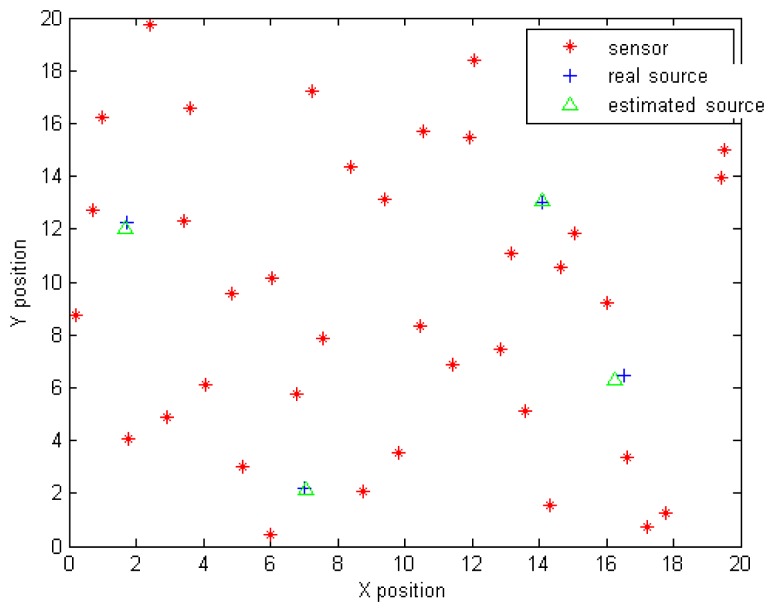
Estimated positions using Bayesian inference and MCMC sampling.

Figure 10 illustrates the true and the estimated positions, which were determined using MCMC slice sampling algorithm in order to solve the Bayesian inference problem. The positions are estimated by computing the mean value. The simulations were carried out using MATLAB on an Intel Core i7-3520M processor (2.90 GHz, 4-MB Cache, Dual-core). The computational time for this approach is 20.35 s. Table 3 shows the estimated parameters using the slice sampling technique.

### 6.2. Convergence Diagnostic

Two common critical issues when using an MCMC sampler in order to estimate the properties of a pdf are, first, how to decide when to stop sampling and use the available samples in order to estimate the characteristics of the posterior distribution of interest, and second, how to determine the number of iterations that correspond to the burn-in and should be discarded [38].

While it is difficult to predict the number of iterations *N_iter_* after which it is safe to stop sampling and the number *N_burn_* of initial samples to be discarded, diagnostic tools can be applied to the output of the MCMC samplers in order to address the convergence problem.

In order to decide if the resulting samples accurately estimate the posterior distribution of interest, we apply in this section a convergence diagnostic to the chain outputted by our sampler. We use the StatLib implementation of the Raftery and Lewis diagnostic (1992) [38]. This test requires as inputs a posterior quantile of interest *q*, an acceptable tolerance *r* for *q* and a probability *s* of being within this tolerance. It outputs, amongst other parameters, the number of iterations *N_iter_* and burn-ins *N_burn_* necessary to satisfy the specified conditions. The diagnostic was run on the resultant Markov chain for *q* = 0.5, *r* = 0.01 and *s* = 0.95, which means we want to measure the 0.5 quantile with an accuracy of 0.01. The output was a total number of iterations *N_iter_* = 2, 655 to be run, of which the first *N_burn_* = 10 samples correspond to the burn-in and should be discarded. Thus, we are 95% sure that the true quantile is within ±0.01 from the corresponding estimated value.

### 6.3. Least Squares Technique

Next, the probabilistic Bayesian approach is compared to the generic least squares optimisation approach.

**Figure 11.**
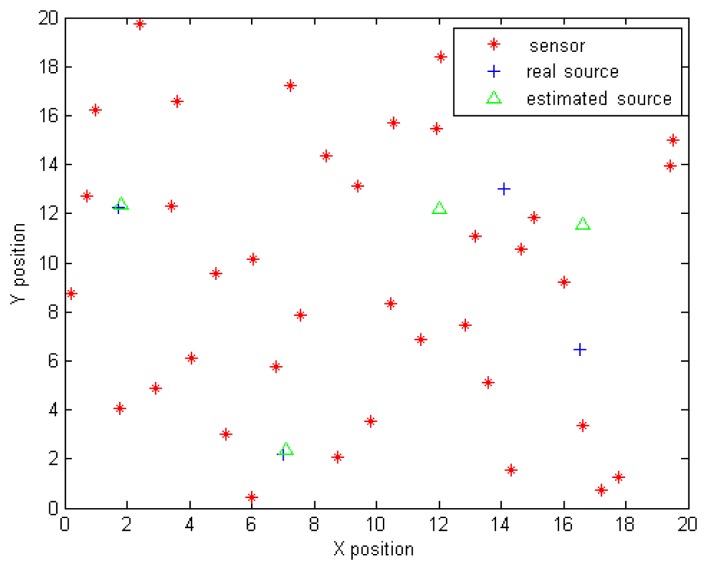
Solution provided by the least squares approach for randomly chosen start parameters.

Looking back at Figure 6, the cost function to be minimised is not convex and has multiple local minima. Recall that this figure illustrates the variation of the functional *J* given by Equation (20) as a function of Source 3 coordinates after fixing the remaining parameters to their exact values. A least squares search algorithm might fall into some local minimum and, thus, diverge from the true global minimum located near Source 3's true position. Figure 11 shows the solution provided by the least squares technique when the vector of parameters is randomly initialised. The algorithm stopped at a local minimum, so the solution provided diverges from the true parameters.

The choice of the initial point for the search algorithm is crucial. If this start point is situated in the restrained convex region around the global minimum (see Figure 6), the least squares approach is likely to converge to the true solution. In order to overcome this problem, we choose the start position parameters to be the positions of the three sensors indicating the greatest concentration measurements. These sensors are likely to be the closest to the sources.

**Figure 12.**
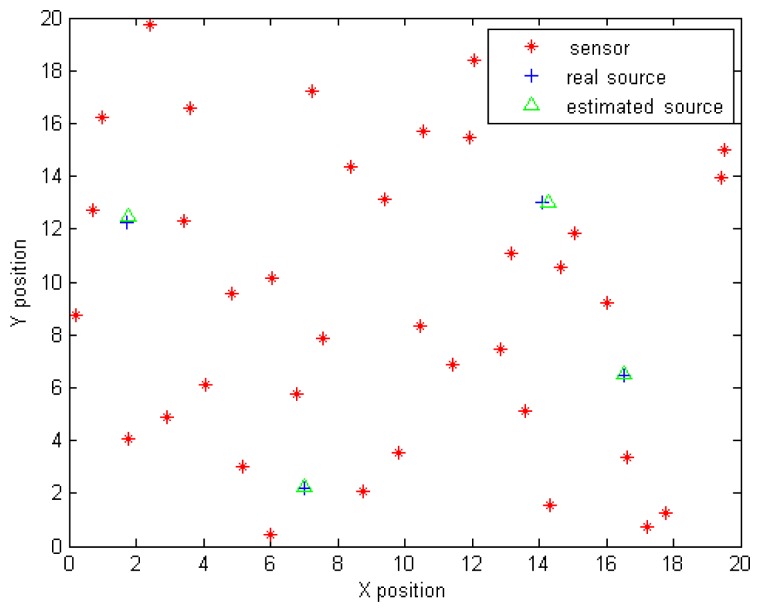
Optimal solution provided by the least squares approach.

Figure 12 illustrates the optimal land mine positions, resulting in minimising the functional *J* given in Equation (20). The start position parameters for the search algorithm are chosen to be the positions of the four sensors, indicating the maximal concentration measurements. The least squares technique provides in this case an accurate estimation of the unknown parameters.

Table 4 groups the optimal results obtained using the least squares technique. The computational time is 6.34 s.

Table 5 groups the mean squared errors on sources positions and emission rates for both approaches.

Both the least squares technique and the Bayesian probabilistic approach offer a similar performance provided an adequate choice of the start point for the optimisation search algorithm. The least squares approach also requires less computational time. However, it is important to note that, as the sensors are randomly deployed, even the ones with the greatest concentration measurements might not always be close enough to the sources, so as to find the global minimum. This optimisation technique is very sensitive to the choice of the initial point. On the other side, the probabilistic Bayesian approach together with an efficient sampling algorithm turns out to be more robust and less sensitive to the choice of the initial sample of the Markov chain.

## 7. Conclusions

While previous work on land mine localisation using sensor networks solves the problem of locating a single source, this paper considers the problem of locating several land mines. It also deals with the more difficult scenario of an unknown number of sources to be characterised. First, the PCA technique is used in order to determine the number of land mines. Second, the inverse problem consisting of locating and estimating the emission rates of the land mines is solved in a probabilistic Bayesian framework. In our simulations, we compare the results obtained using this approach with those provided by the least squares optimisation technique. Both methods localise successfully the sources and provide an accurate estimate of the emission rates of multiple land mines. The main advantage of the probabilistic technique is that, using an efficient sampling scheme, it turned out to be less sensitive to the choice of the initial point of the chain, in contrast with the optimisation technique for which the choice of the start point of the search algorithm is crucial. The probabilistic method also makes it possible to quantify uncertainty on the estimated positions, since a pdf of the unknown parameters is obtained, rather than a single optimal point solution. For future work, a model considering a three-dimensional position for the sources and sensors can be employed. Furthermore, a recent article [39] presents a hierarchical model to find the ground-truth source bases using Nonnegative Matrix Factorisation (NMF) [40]. Since in our approach, PCA was only used to determine the number of sources, the use of source separation techniques (amongst which are PCA and NMF) for source localisation forms an interesting subject of research.

## Figures and Tables

**Table 1.** The minimal number of sensors required in order to detect the true number of sources for the simulation conditions considered in Section 4.

*N*	2	3	4	5	6	7	8

*M*	5	10	16	26	35	48	71

**Table 2.** True parameters.

*i*	(*x_s_i__*, *y_s_i__*)	*Q_i_* [*μg*/*s*]
1	(6.99, 2.18)	8.01
2	(1.73, 12.27)	5.38
3	(16.52, 6.45)	5.87
4	(14.11, 13.01)	9.39

**Table 3.** Estimated parameters using the slice sampling.

***i***	**(*x****_S_i__***,*y****_S_i__***)**	*Q_i_* [*μg*/*s*]
1	(7.06, 2.15)	8.02
2	(1.68, 12.03)	5.44
3	(16.27, 6.27)	5.11
4	(14.10, 13.04)	9.95

**Table 4.** Estimated parameters using the Least Squares search algorithm.

***i***	**(*x****_S_i__***,*y****_S_i__***)**	***Q_i_* [*μg*/*s*]**
1	(6.99, 2.22)	8.53
2	(1.78, 12.49)	5.06
3	(16.54, 6.54)	5.77
4	(14.26, 13.01)	8.98

**Table 5.** Mean squared errors on positions and emission rates.

**Approach**	**Error on**	

	**Position**	**Emission Rate**
Slice sampling	0.040	0.223
Least Squares	0.021	0.137
